# Retraction: Progressive Familial Intrahepatic Cholestasis Type 2 in an Infant: Diagnostic Challenges and Multidisciplinary Management

**DOI:** 10.7759/cureus.r193

**Published:** 2025-10-13

**Authors:** Najeeb Ullah, Somaan Anthony, Arzoo Siddiqi, Nimra Akram, Bismil Afghan

**Affiliations:** 1 Internal Medicine, Rehman Medical Institute, Peshawar, PAK; 2 Internal Medicine, Lady Reading Hospital, Peshawar, PAK; 3 Internal Medicine, Dow University of Health Sciences, Karachi, PAK; 4 Internal Medicine, Jinnah Sindh Medical University, Karachi, PAK; 5 Internal Medicine, Nishtar Medical University, Multan, PAK

The Editors-in-Chief have retracted this article. An investigation by the journal found evidence of authorship manipulation. As a result, the Editors-in-Chief no longer have confidence in the provenance and reliability of the article contents. The authors disagree with the decision to retract.

